# Contrasting community assembly processes structure lotic bacteria metacommunities along the river continuum

**DOI:** 10.1111/1462-2920.15337

**Published:** 2020-12-10

**Authors:** Hyun S. Gweon, Michael J. Bowes, Heather L. Moorhouse, Anna E. Oliver, Mark J. Bailey, Michael C. Acreman, Daniel S. Read

**Affiliations:** ^1^ UK Centre for Ecology & Hydrology Wallingford, Oxfordshire OX10 8BB UK; ^2^ School of Biological Sciences University of Reading Reading RG6 6EX UK; ^3^ Lancaster Environment Centre Lancaster University Library Avenue, Lancaster LA1 4YQ UK

## Abstract

The heterogeneous nature of lotic habitats plays an important role in the complex ecological and evolutionary processes that structure the microbial communities within them. Due to such complexity, our understanding of lotic microbial ecology still lacks conceptual frameworks for the ecological processes that shape these communities. We explored how bacterial community composition and underlying ecological assembly processes differ between lotic habitats by examining community composition and inferring community assembly processes across four major habitat types (free‐living, particle‐associated, biofilm on benthic stones and rocks, and sediment). This was conducted at 12 river sites from headwater streams to the main river in the River Thames, UK. Our results indicate that there are distinct differences in the bacterial communities between four major habitat types, with contrasting ecological processes shaping their community assembly processes. While the mobile free‐living and particle‐associated communities were consistently less diverse than the fixed sediment and biofilm communities, the latter two communities displayed higher homogeneity across the sampling sites. This indicates that the relative influence of deterministic environmental filtering is elevated in sediment and biofilm communities compared with free‐living and particle‐associated communities, where stochastic processes play a larger role.

## Introduction

Lotic habitats (i.e. flowing water) are characterized by high spatiotemporal variability caused by natural factors such as variations in hydrology, temperature, water chemistry and resource availability, including nutrients and both particulate and dissolved organic matter. In addition, anthropogenic activity has extensively modified many river catchments, via changes in land use, channel modification, urban run‐off and sewage discharge (Gessner and Chauvet, 1994; Dodds *et al*., 2000; Biggs *et al*., 2005; Mulholland *et al*., 2008; Boyero *et al*., 2011; Tatariw *et al*., 2013; Atashgahi *et al*., 2015). The longitudinal, lateral and vertical movement of water provides the means for a diverse range of both abiotic and biotic matter to disperse, mix and interact, facilitating complex ecological and evolutionary processes that govern the communities that live within them. Unlike soil bacterial communities where pH and organic matter have frequently been identified as the dominant drivers (Fierer *et al*., 2007), our understanding of lotic microbial ecology still lacks conceptual frameworks for the ecological processes and drivers that shape these communities.

River catchments represent a continuum of interlinked habitats that change considerably in abiotic and biotic character longitudinally, along the river continuum from source to sea (Vannote *et al*., 2008), laterally, via exchanges with the floodplain, and vertically, from free‐flowing water through to the sediment–water interface and the saturated hyporheic zone (Stegen *et al*., 2016). Despite a recent increase in the number of studies of lotic bacteria, many of them have focussed only on individual habitat types and the communities within them, including free‐living (planktonic), particle‐associated planktonic, biofilm or sediment dwelling (Lindström and Bergström, 2005; Battin *et al*., 2007; Crump *et al*., 2007; Wang *et al*., 2013; Gibbons *et al*., 2014; Liu *et al*., 2018; Wisnoski and Lennon, 2020). Consequently, there remain fundamental unanswered questions as to how similar communities found in these contrasting habitats are, what taxa are responsible for any similarities, and what processes are involved in structuring these communities. A previous meta‐analysis comparing studies on lotic habitats indicated that bacterial communities exhibit remarkable consistency within habitat types (Zeglin, 2015). However, lotic habitats are also found along a gradient of contrasting physicochemical conditions, from headwaters to downstream rivers (Vannote *et al*., 2008). As such, it is still unknown whether such consistency is preserved across this river continuum, and whether the processes that govern their community composition are also consistent along this gradient.

Understanding the underlying processes driving their bacterial community assemblages and spatial distribution is becoming increasingly important as the relevance of lotic microbes is recognized in processes such as nutrient recycling (Tatariw *et al*., 2013), global flux of organic carbon into the atmosphere (Battin *et al*., 2008; Raymond *et al*., 2013), persistence and transport of pathogens (Marti *et al*., 2013), biogeochemical processes implicated with climate change (Butman and Raymond, 2011; Raymond *et al*., 2013; Stanley *et al*., 2016), pollution attenuation (Peterson *et al*., 2001; Mulholland *et al*., 2008; Valett *et al*., 2008) and as reservoirs and disseminators of antibiotic resistance (Marti *et al*., 2014).

Two major processes are known to influence how communities are assembled: neutrality‐based stochastic and niche‐based deterministic process (Leibold *et al*., 2004; Lindström and Langenheder, 2012). Neutrality‐based stochastic processes emphasize that communities are neutrally assembled by probabilistic or random dispersal, immigration, or ecological drift. In contrast, niche‐based deterministic processes emphasize that communities are structured by biotic filtering processes such as competition, facilitation and predation, and abiotic filtering where functional differences between individual species play a central role (Nemergut *et al*., 2013). It has been widely recognized that both stochastic and deterministic processes play a role in shaping community assemblages, but their relative contribution varies depending on ecological and environmental conditions (Chase, 2007; Chase and Myers, 2011; Myers *et al*., 2013; Tripathi *et al*., 2018). While recent studies have looked into the relative influence of these underlying processes in different lotic habitat types such biofilms and groundwater (Stegen *et al*., 2012; Wang *et al*., 2013; Veach *et al*., 2016), we addressed this question by examining a range of interacting lotic niches at locations across a river catchment.

In this study, we explore how bacterial community composition and underlying ecological processes differ between four major lotic habitat types (free‐living and particle‐associated plankton in the water column, biofilm on benthic stones and rocks, and sediment) and across a single river catchment (the River Thames in United Kingdom). Bacterial communities across 12 sites, from headwater streams to the main river, alongside a wide range of biotic and abiotic environmental conditions, were investigated with regards to their community composition and structure, as well as the relative contributions of ecological processes that influence the assemblages, ultimately setting a foundation for conceptual frameworks for future research on this topic.

## Methods

### Sampling sites and procedure

**Fig 1:**
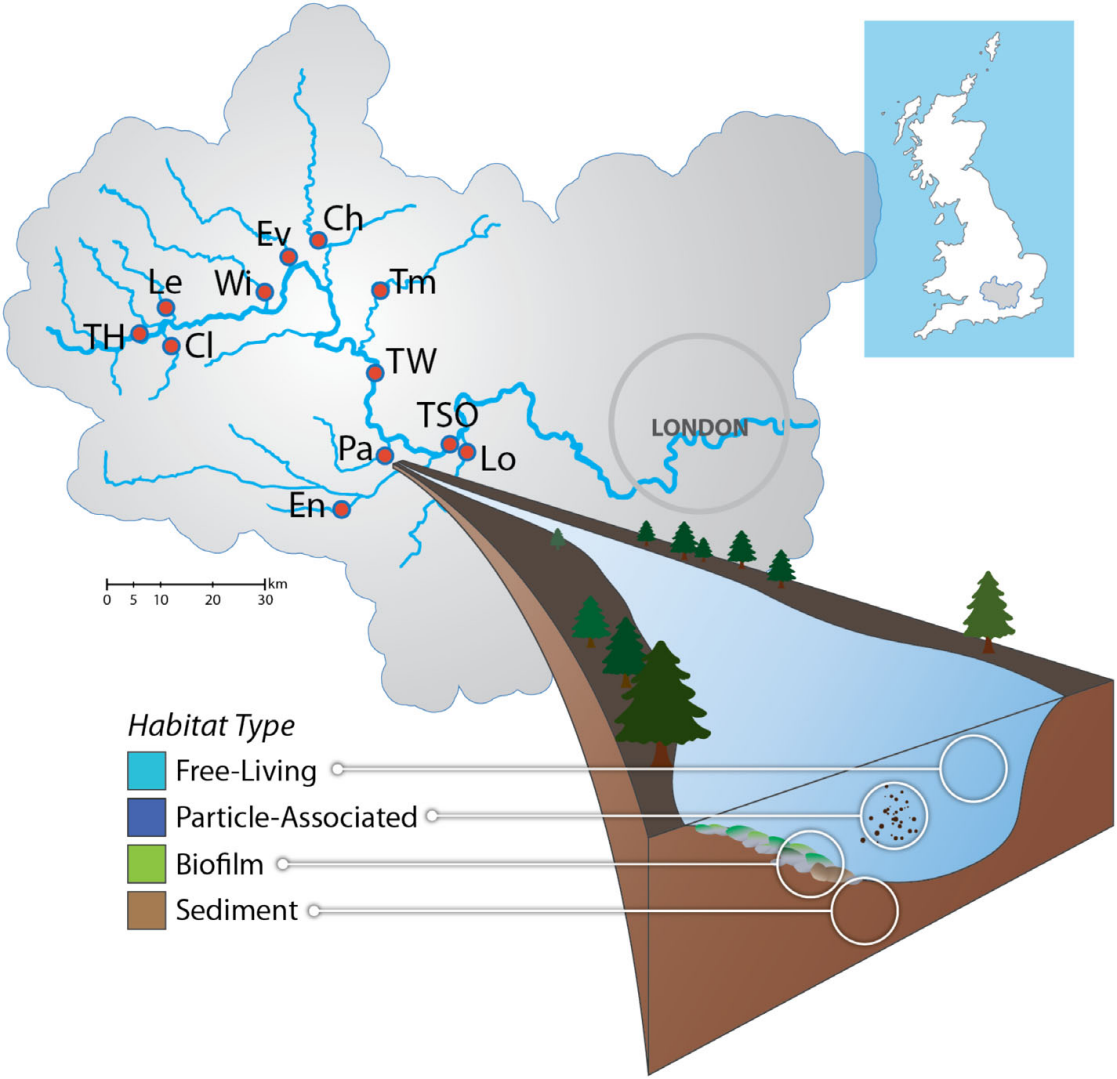
Sampling points and illustrating four habitats in lotic environment. A total of 12 water samples, 111 biofilm samples (between 8 and 10 per site), 60 sediment samples (five per site) were collected (the water samples were divided into free‐living and particle‐associated communities by filtration).

All samples were collected in August 2015 from 12 selected sites across the River Thames basin in southern United Kingdom (Fig. 1, Supplementary Table 7). These sites form part of the UK Centre for Ecology and Hydrology's Thames Initiative research platform (Bowes *et al*., 2018). The selected study sites include three sites along the main channel of the upper and middle River Thames, and nine tributaries representing a wide range of the lotic ecosystem in terms of distance from source, flow, land use, sewage input and cover much of the basin above the tidal limit.

A single water sample was collected from each site using a sterile bucket lowered into the centre of the channel. Samples were transferred to a 1000 ml autoclaved polypropylene bottle and stored in a darkened icebox at 4°C until returned to the laboratory for filtering on the same day. Samples were filtered sequentially through 1.7 μm glass fibre GF/A filters (Whatman, Buckinghamshire, UK) to obtain particle‐associated microbes, and then through a 0.22 μm Durapore membrane filter (Merck Millipore, Watford, UK) to collect free‐living bacteria. Filters were stored at −20°C for DNA extraction. To examine biofilm communities, 8–10 submerged stones exposed to open‐canopy at depths between 10 and 40 cm were randomly collected from each site by hand using sterile gloves. The stones were placed in sterile Whirl‐Pak™ bags and stored in an icebox at 4°C until returned to the laboratory for processing. For each stone, approximately a 5 cm × 5 cm area exposed to the water column was thoroughly brushed with 40 ml of deionized water to remove the biofilm, using a disposable toothbrush. These had been pre‐sterilized in 10% sodium hypochlorite and rinsed in MQ water. After brushing, the liquid was poured into a 50 ml centrifuge tube and centrifuged at 5000*g* for 10 min. The supernatant was discarded, and the pellet was retained and stored at −20°C for DNA extraction. Five sediment sample cores were collected from each site using a Corning™ Falcon 50 ml Conical tube. The samples were put into an icebox in an upright position and were immediately placed in a −80°C freezer when returned to the laboratory. Frozen cores were then cut with a hacksaw (sterilized with 10% sodium hypochlorite and flame) to remove the top 2 cm of the sediment to ensure that biofilm exposed to water column was excluded. From the remaining top layer, 0.25 g of sediment was used for the subsequent DNA extraction.

### DNA extraction and sequencing

DNA was extracted using the PowerSoil‐htp 96 Well Soil DNA Isolation Kit (Mobio Laboratories, Carlsbad, USA) using the manufacturer's protocol. Polymerase chain reaction (PCR) negative controls consisting of extraction and PCR blanks were also processed to remove likely potential contaminants in subsequent data processing (Salter *et al*., 2014). Approximately 20–30 ng of template DNA was amplified using Q5® high‐fidelity DNA polymerase (New England Biolabs, Hitchin, UK) each with a unique dual‐index barcode primer combination (Kozich *et al*., 2013). Individual PCR reactions employed 25 cycles of an initial 30 s, 98°C denaturation step, followed by annealing phase for 30 s at 53°C and final extension step lasting 90 s at 72°C. Primers were based upon the universal primer pair 341F (5′‐CCTACGGGAGGCAGCAG‐3′) (Muyzer *et al*., 1993) and 806R (5′‐GGACTACHVGGGTWTCTAAT‐3′) (Caporaso *et al*., 2011). An amplicon library consisting of approximately 550 bps amplicons spanning the V3 to V4 hypervariable regions of the 16S rRNA gene was sequenced at a concentration of 5.4 pM with a 0.6 pM addition of PhiX on an Illumina MiSeq platform (V3 chemistry, 600 cycles).

### Data processing

Sequenced paired‐end reads were joined using PEAR v.0.9.10 (Zhang *et al*., 2014), quality filtered using FASTX tools v.0.0.14 (fastq_quality_filter, kept reads that have at least 80% of bases with a quality score of 30 or more) (Gordon and Hannon, 2010) and chimeras were identified and removed with VSEARCH_UCHIME_REF v.2.1.2 (Rognes *et al*., 2016) using Greengenes Release 13_8 (97%) (Larsen *et al*., 2006). Singletons were removed and the resulting sequences were clustered into operational taxonomic units (OTUs) with VSEARCH_CLUSTER_FAST v.2.1.2 (Rognes *et al*., 2016) at 97% sequence identity (Tindall *et al*., 2010). Representative sequences for the OTUs were taxonomically assigned with the RDP Classifier v.2.12 with the bootstrap threshold of 0.8 or greater (Wang *et al*., 2007) using the Greengenes Release 13_8 (FULL) (Larsen *et al*., 2006) as a reference. Unless stated otherwise, default parameters were used for the steps listed. Where multiple replicate samples were collected from a site, ≥75% prevalence filter was used to select OTUs that best represent the site and their abundances were then averaged into a single sample. The OTU table was subsequently rarefied down to 24 007 sequences per sample to address variability in sampling depth (Weiss *et al*., 2015) and to ensure that the number of reads used for each of the 48 samples is the same for downstream analysis. Low‐abundance OTUs below 0.01% abundance (Bokulich *et al*., 2013) and those that belong to ‘chloroplasts’ were removed. The representative sequences for the OTUs were aligned using SSU‐ALIGN v.0.1 (Nawrocki, 2009) and FastTree v.2.1.9 (Price *et al*., 2009) with a GTR + G substitution model used on the resulting alignment to generate a phylogenetic tree to infer phylogenetic relationships among the OTUs.

### Statistical analysis

To assess differences in community compositions, the Bray–Curtis dissimilarities were determined which quantify compositional differences based on relative abundances of OTUs. One‐way ANOVA and Tukey HSD (Honestly Significant Difference) tests, Shannon index, Pielou's evenness index, Whittaker's beta diversity, distances of group members to the group centroid, non‐metric multidimensional scaling (Bray–Curtis dissimilarity with 999 randomisations) and permutational multivariate analysis of variance test (PERMANOVA) were performed using the package Vegan v.2.4‐1 (Oksanen *et al*., 2007). Values were Bonferroni‐corrected where applicable.

### SPEC‐OCCU plots

The 500 most abundant OTUs were selected from the OTU table for each habitat, and from the table, specificity and occupancy (or otherwise known as fidelity) were calculated as per Dufrene and Legendre (1997). Specificity is defined as the mean abundance of species (*S*) in the samples of habitat (*H*); and occupancy is defined as the relative frequency of occurrence of *S* in the samples of *H* (See Eq. 1).

(1) $$\begin{array}{l} \text{Specificity}=\frac{{\text{Nindividuals}}_{S,H}}{{\text{Nindividuals}}_{S}}\\ \text{Occupancy}=\frac{{\text{Nsites}}_{S,H}}{{\text{Nsites}}_{H}} \end{array}$$

Nindividual_S,H_ is the mean number of individual OTU *S* across all samples of habitat *H*, while Nindividual_S_ is the sum of the mean number of individual *S* over all habitats; Nsites_S,H_ is the number of samples in *H* where *S* is present, while Nsites_H_ is the total number of samples in *H* (Dufrene and Legendre, 1997). These two metrics were subsequently used as the axes in SPEC‐OCCU plots.

### Phylogenetic turnover

We implemented a previously developed null modelling approach to infer community assembly processes (Stegen *et al*., 2013; Wang *et al*., 2013; Dini‐Andreote *et al*., 2015). We used Phylocom v.4.2 (Webb *et al*., 2008) to compare phylogenetic turnover between the habitats by calculating the β‐Nearest Taxon Index (βNTI) from the phylogenetic tree. For each pair of communities within each habitat, βNTI was generated using the independent swap null model (Gotelli and Entsminger, 2003) to generate null communities (999 permutations). The βNTI is a standardized effect size measure that expresses the difference between observed between‐community mean‐nearest‐taxon‐distance and the mean of null distribution in units of standard deviation (Stegen *et al*., 2012; Stegen *et al*., 2015). A mean βNTI value less than −2 or greater +2 across all pairwise comparisons suggests deterministic processes while a value between −2 and + 2 is considered to signify the influence of stochastic assembly (Stegen *et al*., 2012; Zhou and Ning, 2017).

## Results

### Sequences and OTUs

The 195 samples from the 12 sites across the Thames basin generated a total of 22 292 615 paired‐end raw sequences, which were reduced to 20 252 671 post‐processed sequences with an average of 77 895 sequences per sample. After the rarefaction, 4,608,494 sequences remained that clustered into 15 962 OTUs (3564, 5330, 8606 and 10 124 OTUs for free‐living, particle‐associated, biofilm and sediment bacterial communities respectively).

### Bacterial community diversity and composition

**Fig 2:**
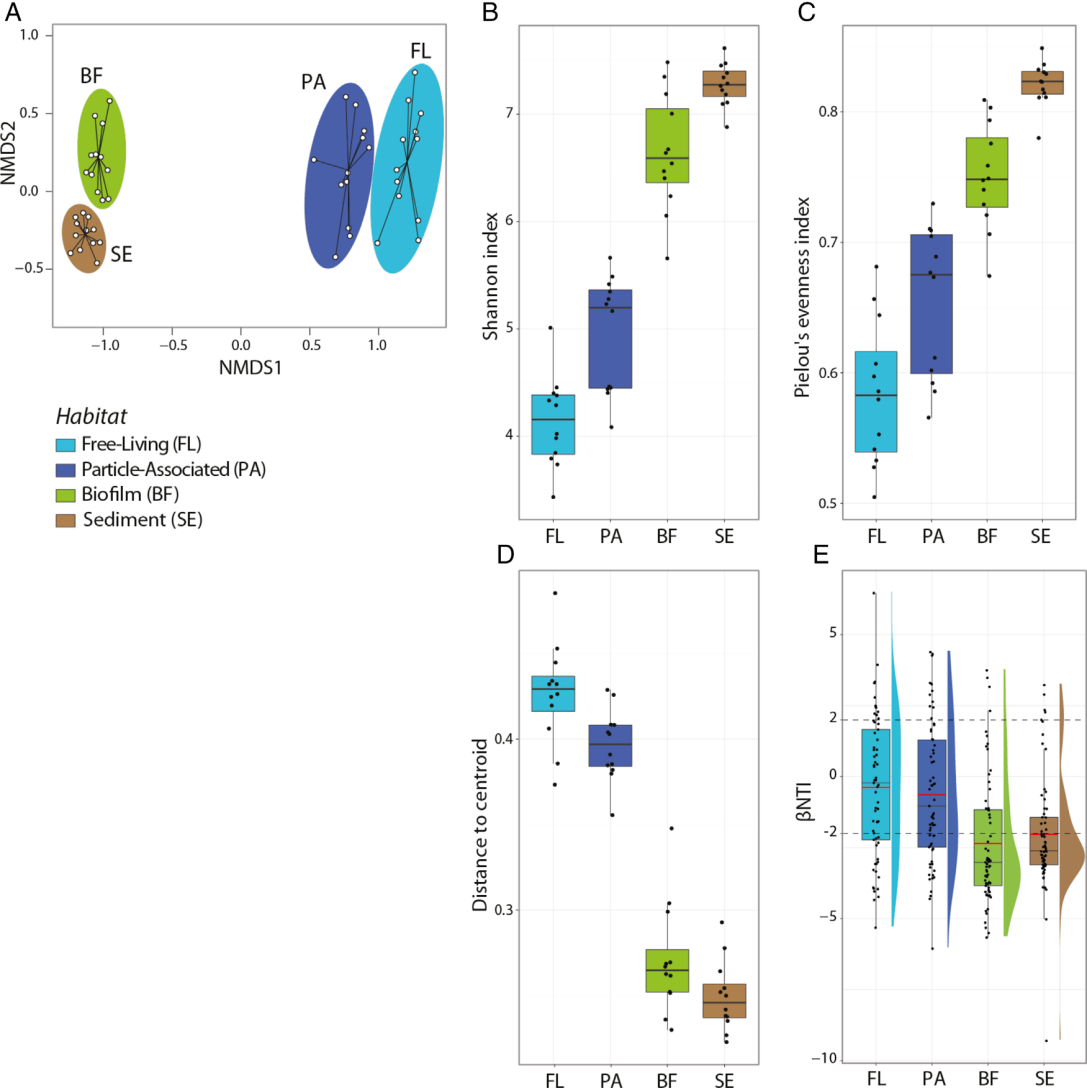
A. Non‐metric multidimensional scaling (NMDS) resulting from Bray–Curtis dissimilarity matrices exhibited distinct differences in community composition between all four habitat types (PERMANOVA between all pairs: *p*‐value <0.005). B. Increase in Shannon diversity index from FL, PA and through BF and SE. C. Increase in Pielou's evenness indices again from FL to SE. D. Distance to centroid in multivariate homogeneity of group variance analysis for lotic bacterial communities in four habitat types (A post hoc Tukey's HSD: marginal significance between FL and PA, *p* = 0.038; between BF and SE, *p* = 0.225, all other pairs were significant, *p* < 0.001; See Supplementary Table S2c). E. β‐Nearest Taxon Index (βNTI) for all pairs of communities within each habitat. A mean βNTI (red lines: −0.3819, −0.6356, −2.353, −2.034 for free‐living, particle‐associated, biofilm and sediment habitats respectively) across all pairwise comparisons that is greater than +2 or less than −2 (dotted lines) as are seen in benthic communities (biofilm and sediment) suggests the influence of deterministic processes in microbial assembly.

An NMDS ordination plot highlighted a distinct segregation between communities from all four habitat types (Fig. 2A, PERMANOVA, *p* < 0.005). There was greater segregation between the planktonic (free‐living and particle‐associated) and benthic (biofilm and sediment) communities (see Supplementary Table S1). Diversity (Fig. 2B) and evenness (Fig. 2C) also varied along these habitats, where both indices increased from free‐living, particle‐associated, through biofilm to sediment (ANOVA, Shannon: DF = 3, F (Larsen *et al*., 2006; Tatariw *et al*., 2013) = 127.03, *p* < 0.0001; Evenness: DF = 3, F (Larsen *et al*., 2006; Tatariw *et al*., 2013) = 62.167, *p* < 0.0001; A post hoc Tukey's HSD *p* < 0.01—see Supplementary Tables S2a & S2b). There was a significant difference in beta diversity between the planktonic and benthic communities, where the benthic communities were much more homogenous than the planktonic communities (Fig. 2D).

### Bacterial community composition and structure at the phylum level

**Fig 3:**
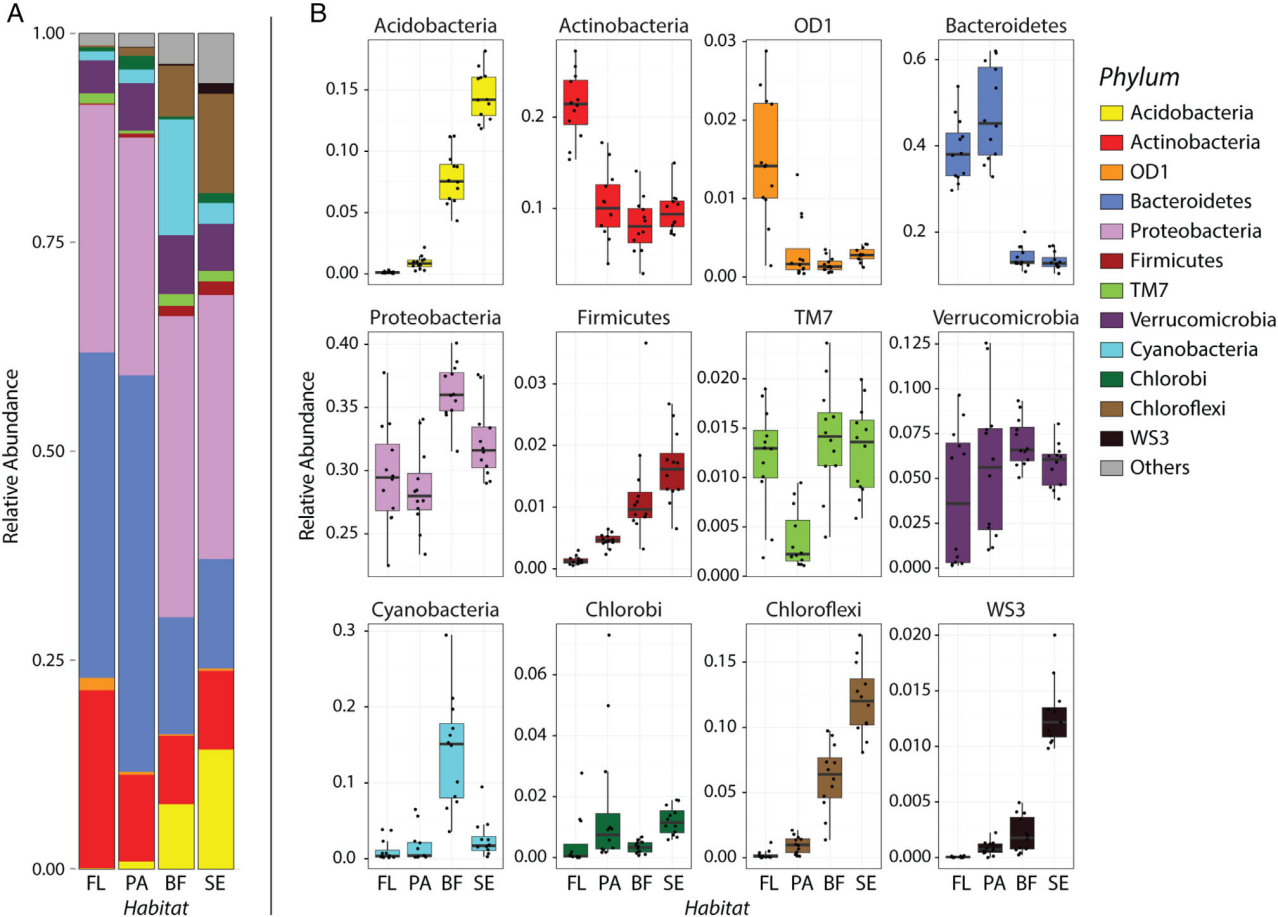
(A) Relative abundances of bacterial phyla in the four lotic habitat types. B. Relative abundances of 12 most abundant bacterial phyla.

At the phylum level, the most abundant and common taxa across all habitat types were Bacteroidetes, Proteobacteria, Actinobacteria and Verrucomicrobia (Fig. 3A). The proportion of Bacteroidetes was significantly higher (a post hoc Tukey's HSD *p* < 0.000—see Supplementary Table S3a) in planktonic habitats (free‐living: 38.9%, particle‐associated: 47.5%) than the benthic habitats (biofilm: 14.1%, sediment: 13.3%), and Actinobacteria were relatively more abundant in free‐living. Although Verrucomicrobia were present in similar relative abundance in all habitats, a greater variance in the planktonic communities was observed. Cyanobacteria were significantly more abundant in biofilm samples (a post hoc Tukey's HSD *p* < 0.000—see Supplementary Table S3a). Notably, the proportion of Acidobacteria and Chloroflexi showed an increasing trend from free‐living, particle‐associated, biofilm to sediment. OD1 was statistically more abundant in the free‐living habitat (a post hoc Tukey's HSD *p* < 0.000—see Supplementary Table S3b). While Firmicutes and WS3 were not abundant in all communities they showed a clear increasing trend in relative abundance from free‐living, particle‐associated, biofilm to sediment (Fig. 3B).

### Distribution and overlap of the abundant OTUs across lotic locations

**Fig 4:**
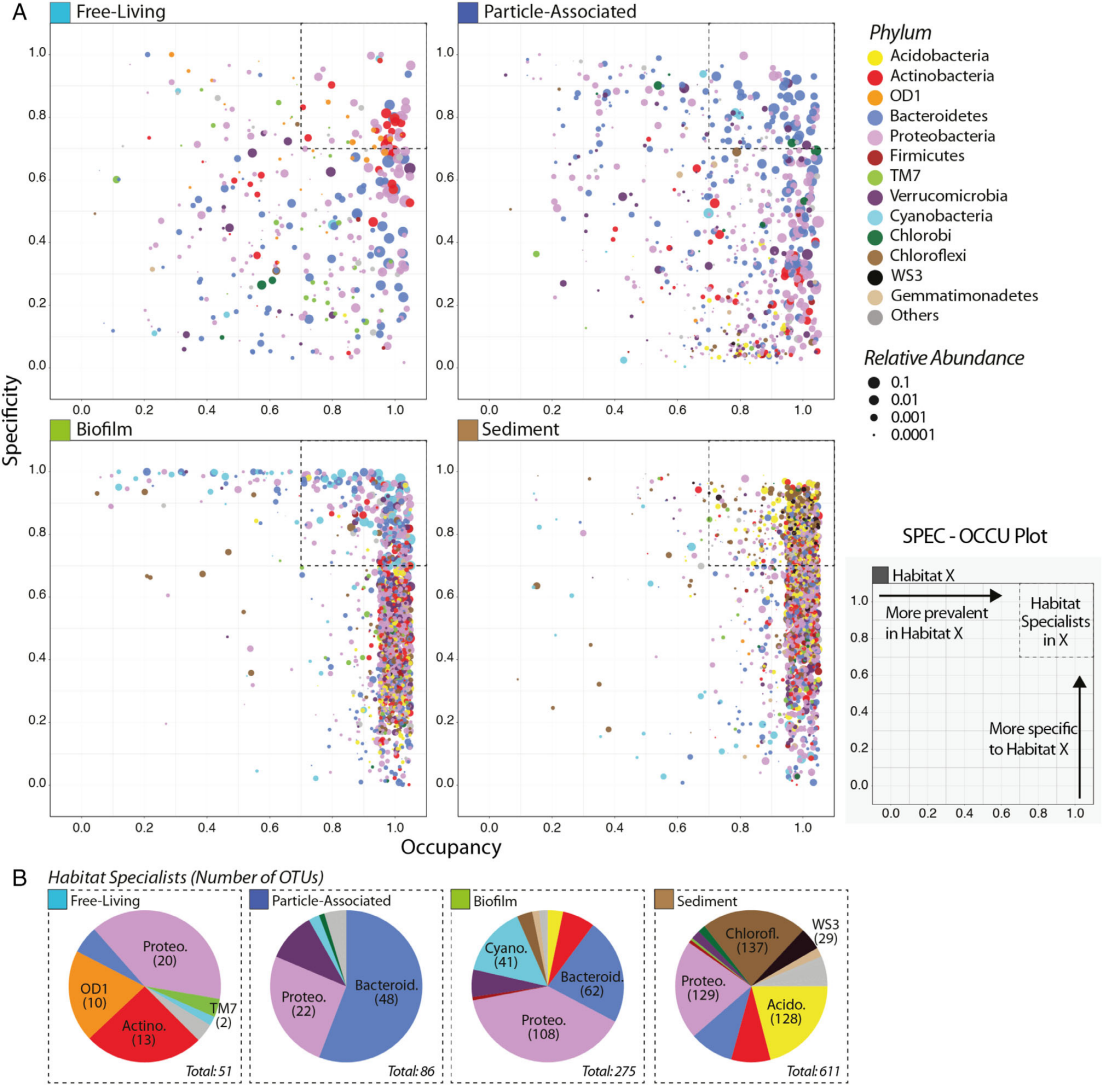
(A) The SPEC‐OCCU plots show 500 most abundant OTUs in each habitat type; the *x*‐axis represents occupancy, i.e. how well an OTU is distributed across all 12 sites; and the *y*‐axis represents specificity, i.e. whether they are also found in other habitats. B. Pie charts showing the number of OTUs representing specialists in each habitat (See Supplementary Table S6 for the list of these specialists).

To inspect how OTUs from each habitat type are spread across sites and also how specific they are to their habitat, specificity and occupancy were calculated for each OTU (Eq. 1) which were then projected onto a plot (SPEC‐OCCU plot, Fig. 4A). As indicated by the spread of OTUs across the *x*‐axis (occupancy), OTUs from free‐living communities displayed highly varied occupancy (i.e. fewer were present in all sites), while the majority of OTUs from sediment communities exhibit remarkably homogenous occupancy at sites across the river catchment. To find specialist species attributable to each habitat type, we selected OTUs with specificity and occupancy greater or equal to 0.7 (dotted boxes in Fig 3A and B), i.e. they are specific to a habitat and common in their habitat in most sites. The number of these specialist OTUs differed significantly between the habitats with an increasing trend in terms of richness from free‐living (51 OTUs represent), particle‐associated (86 OTUs), biofilm (275 OTUs) to sediment (611 OTUs) representing 5.4%, 5.4%, 8.5% and 7.4% of the total sequences respectively. Proteobacteria and Bacteroidetes were found in all four habitat specialist groups while notable free‐living specialists included OTUs from OD1 and TM7. Particle‐associated specialists were mostly Bacteroidetes and Proteobacteria, with no Actinobacteria found to be both specific and common in this habitat type. Notable biofilm specialists were Cyanobacteria, and sediment specialists included OTUs from Acidobacteria, Chloroflexi and WS3.

The number and taxonomic composition of the OTUs shared between different sets of habitats were also compared (Fig. S1). Overall, 5735 OTUs were found in a single habitat (3045 OTUs in sediment, 1419 OTUs in biofilm, 646 OTUs in free‐living and 625 OTUs in particle‐associated) while 1279 OTUs occurred in all four habitats. The habitats with the highest number of shared OTUs were biofilm and sediment (3103 OTUs). Sediment, free‐living and particle‐associated communities had the least shared OTUs (145 OTUs), followed by sediment and free‐living (150 OTUs), highlighting the distinctness of these habitats.

### The influence of deterministic or stochastic processes

To infer the relative influence of deterministic and stochastic processes in each habitat type, we examined βNTI. The mean βNTI values were −0.3819, −0.6356, −2.353 and −2.034 for free‐living, particle‐associated, biofilm and sediment habitats respectively (Fig. 2E). This result suggests that the relative influence of deterministic processes in community dynamics are much higher in benthic habitat types (sediment in particular) compared with planktonic habitats, where stochastic processes play a more significant role.

## Discussion

Recently there has been increased interest in the processes that structure lotic microbial communities (Read *et al*., 2015; Ruiz‐González *et al*., 2015; Savio *et al*., 2015; Niño‐García *et al*., 2016; Henson *et al*., 2018), partly due to the recognition that streams and rivers act as ‘active pipes’ that process nutrients and organic matter rather than passively channelling them from land to sea (Cole *et al*., 2007). As a result, lotic environments are now seen as sites of intense biological activity that play a major role in global biogeochemical cycles (Raymond *et al*., 2013; Tranvik *et al*., 2018). As microbes are primarily responsible for these cycles, understanding the ecological controls over community composition is a necessary first step to developing a more complete understanding of both the ecology and the biogeochemistry of flowing water. However, this is complicated by the fact that lotic environments are made up of a number of distinct habitats that occur across a gradient of physicochemical conditions from headwaters to downstream, and our knowledge of how these communities are structured relative to one another and how much ecological exchange occurs between these habitats is lacking.

### Community composition across habitats

Our results demonstrate that there is a distinct segregation in bacterial community composition between habitat types (free‐living, particle‐associated, biofilm and sediment), and this pattern is consistent across the catchment (from tributaries to the main river channel or the River Thames) despite considerable variation in water physicochemical water quality conditions between sites (Supplementary Table S1). Habitat types were distinct from one another, not only in overall community composition but also in diversity, evenness and variance in community composition within a habitat. These results suggest that, despite the extensive physical and chemical environmental gradients between sites across the river catchment, each habitat type provides sufficiently unique conditions to facilitate communities to be assembled that are consistent in composition and structure according to their habitat type.

Our observations of the community composition of each habitat are largely in agreement with previous studies focussing on individual lotic habitats. For example, across all sites, OTUs belonging to the phyla Actinobacteria and OD1 (also referred to as Parcubacteria) were significantly enriched in the free‐living or planktonic community (Fig. 2B), agreeing with previous findings examining lotic bacterioplankton (Peura *et al*., 2012; Jackson *et al*., 2014; Read *et al*., 2015; Savio *et al*., 2015), where these taxa, along with the Proteobacteria are frequently characteristic of freshwater free‐living planktonic communities. Furthermore, it has been suggested that members of the OD1 exhibit either a free‐living or symbiotic lifestyle due to their reduced genome size and lack of biosynthetic capabilities (Nelson and Stegen, 2015). If symbiosis is the predominant lifestyle in lotic OD1, the symbiotic relationship is likely to be with small commensal/symbiotic/parasitic organisms, present as free‐living plankton and able to pass through a 1.7 μm filter (the ‘free‐living’ fraction in this study). Future studies would need correlative techniques such as visualization with FISH or SEM, or flow sorting and genomic investigation to give a further insight into these interactions. The second characteristic group of the free‐living fraction, the Actinobacteria, have frequently been identified as a dominant member of free‐living freshwater communities, both in lakes (Allgaier and Grossart, 2006; Neuenschwander *et al*., 2018) and in the lower reaches of rivers (Read *et al*., 2015; Savio *et al*., 2015; Henson *et al*., 2018). It has been suggested that they are well suited to a free‐living planktonic lifestyle due to their small size, metabolic versatility and the presence of rhodopsin pigments that can be used to harvest sunlight for energetic and photosensory functions (Newton *et al*., 2007; Sharma *et al*., 2009; Ghai *et al*., 2014).

Suspended particles play a crucial role in freshwater environments as microhabitats for cell attachment and as sources of labile carbon (Rieck *et al*., 2015; Cordero *et al*., 2016). Bacterial communities found on particles have previously been shown to have a high degree of specialization (Crump *et al*., 1999; Rösel *et al*., 2012), and whilst the particles are planktonic, the attached lifestyle of particle‐associated microbes allows for higher levels of cell–cell interactions between cells (Amalfitano *et al*., 2017) and rates of activity (Ghiglione *et al*., 2009). Our results confirm that communities on suspended particles were distinct from the planktonic, biofilm and sediment communities (Fig. 3A) in community composition, and also in diversity, evenness and variance. In contrast to Niederdorfer *et al*. (2016) who found that experimentally manipulated communities associated with suspended aggregates had a higher richness and Shannon's Index when compared with biofilms, we found that the mobile free‐living and particle‐associated communities were consistently less diverse than the fixed sediment and biofilm communities. The reasons for this are not clear, although it may be due to the different lotic systems studied, or reflect the difficulties in using experiments to represent natural systems (Carpenter, 1996). Particle‐associated communities in the River Thames were characterized by members of the phylum Bacteroidetes, which have previously been highlighted as being particle‐associated in aquatic environments (Fernández‐Gómez *et al*., 2013; Milici *et al*., 2017), and have been shown to play an important role in the degradation of complex biopolymers (Kirchman, 2002).

Biofilm communities in the Thames catchment were enriched in Acidobacteria, Proteobacteria, Chloroflexi and Cyanobacteria when compared with the planktonic habitats, which is consistent with previous studies on the major constituents of freshwater biofilms (Battin *et al*., 2016). Chloroflexi have previously been identified as playing a critical role in carbon cycling, and vary in metabolic lifestyle including photoautotrophs, fermentative species, organohalide respiring species and aerobic thermophiles (Hug *et al*., 2013). Biofilms are considered to be a major component of lotic food webs, as key sites of enzymatic activity that play a role in organic matter cycling, ecosystem respiration and primary production (Battin *et al*., 2016). Their position at the interface between the water and sediment means that they have the potential to mediate both the exchange of chemicals as well as the exchange of cells between these habitats. Biofilm communities were significantly different in composition to those found in the free‐living and particle‐associated fractions, as well as containing a more diverse community that was more even in terms of species abundance and homogeneous across sites.

Sediment communities were characterized by OTUs from the phyla Acidobacteria, Chloroflexi and Proteobacteria. Acidobacteria have previously been shown to be particularly abundant in soils and sediments (López‐García *et al*., 2003; Janssen, 2006; Barns *et al*., 2007) and recent genomic and metagenomics studies indicate that their successful adaptation to the sediment environment could be due to their ability to produce high‐affinity transporters, exploit a wide variety of carbohydrates as substrate and produce secondary metabolites as well as being resistant to antibiotics (Ward *et al*., 2009; Kielak *et al*., 2016). It is worth noting that the sediment community features a unique phylum WS3, also known as the candidate phylum ‘*Latescibacteria*’. This is consistent with previous studies on freshwater sediment (Morrison *et al*., 2017; Guan *et al*., 2018). Another study derived from single‐cell amplified genomes of WS3 indicates their heterotrophic and strictly fermentative lifestyle and suggested that it may play a crucial role in the turnover of algal detritus (Farag *et al*., 2017).

One group, the Proteobacteria, was present in a high abundance across all lotic habitats (Fig. 3) and habitat specialists were found from this group in all habitats (Fig. 4). The ubiquity of Proteobacteria in freshwater has previously been identified by Graham *et al*. (2017), in a study of free‐living and attached microbes in the hyporheic zone of the Columbia River in the United States. Proteobacteria contain members with diverse energy requirements, and the ability to assimilate a range of substrates such as sulfate, nitrate, and inorganic C, as well as diverse organic C compounds (Gupta, 2000; Kersters *et al*., 2006). This suggests that they may have members who can thrive in the varying conditions found in both planktonic and sessile lifestyles.

### OTU‐level habitat specificity and overlap

Since lotic ecosystems are characterized by constantly mixing water, the opportunities for the exchange of taxa between habitats are high. Due to this unique characteristic of lotic environments, it has been speculated that many aquatic bacteria possess a complex lifestyle with the ability to alternate between different stages to take advantage of available nutrients or other environmental factors such as light and symbionts (Grossart, 2010). We found many OTUs with low specificity to a single habitat type; for example, 1279 OTUs were found across all habitats, and the highest number of OTUs (3103) were found exclusively in both biofilm and sediment (Supplementary Fig. 1). However, there are also OTUs that are both highly specific to one habitat type and common across all sites within the catchment (dotted box in Fig. 4A). The sediment had the highest number of *habitat specialist* OTUs (611 OTUs), while biofilm, particle‐associated and free‐living habitat types had 275, 86 and 51 OTUs respectively.

### Within habitat beta‐diversity

Planktonic communities, both free‐living and particle‐associated, are generally expected to be heterogeneous in both space and time, as continuous inflow of water from a wide range of terrestrial sources allows a diverse pool of source communities to be assembled (Ruiz‐González *et al*., 2015). Despite this, a number of recent catchment‐scale studies have highlighted that bacterial communities can exhibit a predictable gradient in community composition from the headwaters to the downstream river, driven by the relative importance of terrestrial inputs and species sorting along the river continuum (Read *et al*., 2015; Savio *et al*., 2015; Niño‐García *et al*., 2016; Henson *et al*., 2018). Although this study was not designed to capture the full headwater to downstream gradient, sites were chosen to encompass a wide range of physicochemical parameters and hydrology types across the catchment (Supplementary Table S1). We observed that although the free‐living community had the lowest diversity and evenness it also had the highest variance across the sites (Figs 2B–D), possibly reflecting the relative importance of immigration or ‘mass effects’ (Leibold *et al*., 2004) from the surrounding catchment in structuring these communities (Ruiz‐González *et al*., 2015). Due to the importance of mass effects in structuring lotic bacterioplankton it is not clear which members of the community are simply ‘passing through’ and play no role in the functioning of the ecosystem (other than as a potential source of nutrients) and which members are proliferating and adapted to the freshwater niche.

Compared with the planktonic habitats, both the biofilm and sediment communities show significantly higher homogeneity (Fig 2A and D) across the sampling sites. This is apparent in the SPEC‐OCCU plots (Fig. 4A), where the majority of the OTUs have high occupancy, meaning that most of them are also found in all other sites across the catchment. These results indicate that the sessile (biofilm and sediment) environments are highly selective for a large and stable core community, despite differences in the overlaying water conditions (Supplementary Table S4). It is not immediately obvious how the biofilm and sediment communities are able to display such spatial homogeneity. We did not investigate the physical and chemical characteristics of the sediment or biofilms themselves, so further work is needed to find out if these are responsible for buffering the varying nutrient levels in the overlaying water, and thus assembling similar communities, or whether other factors, possibly related to an attached lifestyle, are responsible.

### Community assembly processes

Aquatic systems, and the communities they contain, are dynamic, and the movement of microbes along river networks, as well as between habitats within the river channel, represents a challenge to developing an understanding of the processes responsible for community assembly in these systems. The deterministic theory asserts that biotic and abiotic filtering play a major role in structuring species composition, whereas stochastic theory emphasizes that communities are neutrally assembled by probabilistic dispersal, ecological drift or historical inertia and neglects the functional differences between species (Hubbell, 2001). Both indices indicate deviations from null model expectations across temporal and spatial environmental gradients and allowed us to infer the relative influences of stochastic and deterministic processes between habitats and samples. The resulting βNTI distributions and their means (Fig. 2E) suggest that the relative influence of deterministic environmental filtering is elevated in sediment and biofilm communities compared with free‐living and particle‐associated communities, where stochastic processes play a larger role. We speculate that deterministic processes such as niche filtering are responsible for creating consistent communities in the sediment and biofilm habitats (Fig. 2A and D). This is supported by the higher degree of habitat specialization observed among the OTUs in sediment and biofilm communities, indicating that the unique properties of these environments (and potentially lower rates of mass effects) are responsible for structuring these communities. Previous studies have indicated that biofilms have structural resilience and possess resistance to environmental and chemical influences (Mah and O'Toole, 2001; Donlan and Costerton, 2002; Hall‐Stoodley *et al*., 2004). Sediment has also been demonstrated to contain resilient structures kept intact by internal adhesives such as biofilms and supracellular ropes (Garcia‐Pichel and Wojciechowski, 2009; Vignaga *et al*., 2013; Battin *et al*., 2016), potentially contributing to a high degree of environmental filtering in this environment.

Both the planktonic habitats had a mean βNTI between −2 and +2, indicating that the stochastic process plays an important role in structuring these environments. This is perhaps unsurprising, given that surface water originates from a variety of sources, including groundwater, surface runoff, sewage inputs and connections to manmade water sources such as canals and reservoirs. A study in a relatively un‐impacted catchment identified a clear trend in the influence of terrestrial inputs (Ruiz‐González *et al*., 2015), thus highlighting the important role of the surrounding terrestrial landscape in shaping freshwater ecology (Reid *et al*., 2018).

## Conclusions

Our results indicate that there are distinct differences in community structure and composition between the four major lotic habitat types, with substantial contrast in ecological processes shaping their community assemblages. The high dispersal and invasion ability of bacteria in a uniform environment can lead to the ubiquity of communities across space and time (Van der Gucht *et al*., 2007; Urban and De Meester, 2009). We speculate that the constant supply of migrating individuals in the water column, alongside the sufficiently stable habitats of sediment and biofilm environments, results in the remarkably heterogeneous microbial communities found in each habitat type.

## Author Contributions

HSG and DSR conceived and designed the study. HSG, HLM, MJBo and DSR performed the fieldwork. HSG performed the molecular work in the laboratory. AEO performed DNA quantification and sequencing. HSG performed the sequence analysis, statistical analyses, interpreted the statistical findings and wrote the manuscript. MJBa, MCA and DSR contributed to the preparation of the manuscript.

## Supporting information


**Supplementary Figure 1** Upset chart showing overlap in all OTUs identified in each habitat type. Numbers of OTUs shared between different sets of habitats are indicated in the top bar chart and the specific habitats in each set are indicated with solid points below the bar chart. Total number of OTUs for each habitat is indicated on the left. Figure generated using Upset R package (Lex *et al*., 2014). Do note that the bar chart is not showing relative abundances.

**Supplementary Table 1** PERMANOVA analyses produces a p‐value for significance and the R2 value, which indicates the amount of variation attributed to a specific treatment within a model. The R2 values indicate greater differences between the planktonic communities (Free‐Living and Particle‐Associated) and benthic communities (Biofilm and Sediment).

**Supplementary Table 2** Tukey's HSD on diversity indices (Tukey multiple comparisons of means, 95% family‐wise confidence level)

**Supplementary Table 3** Tukey's HSD on the differences in relative abundances of selected phyla between four different habitats (Tukey multiple comparisons of means, 95% family‐wise confidence level)

**Supplementary Table 4** Supporting information

**Supplementary Table 5** Supporting information

**Supplementary Table 6** Supporting information

**Supplementary Table 7** Supporting information

## Data Availability

The raw sequence data reported in this study have been deposited in the European Nucleotide Archive under the accession number PRJEB29699. The relevant barcode information for each sample is shown in Supplementary Table S5.
