# Supplementary material for: Contrasting community assembly processes structure lotic bacteria metacommunities along the river continuum
Source: Environ Microbiol. 2020 Dec 10;23(1):484–98. doi: 10.1111/1462-2920.15337 (PMC7898806; doi:10.1111/1462-2920.15337)
Supplement: Supplementary file 1 — Supplementary Figure 1 Upset chart showing overlap in all OTUs identified in each habitat type. Numbers of OTUs shared between different sets of habitats are indicated in the top bar chart and the specific habitats in each set are indicated with solid points below the bar chart. Total number of OTUs for each habitat is indicated on the left. Figure generated using Upset R package (Lex et al., 2014). Do note that the bar chart is not showing relative abundances. [file EMI-23-484-s001.docx]

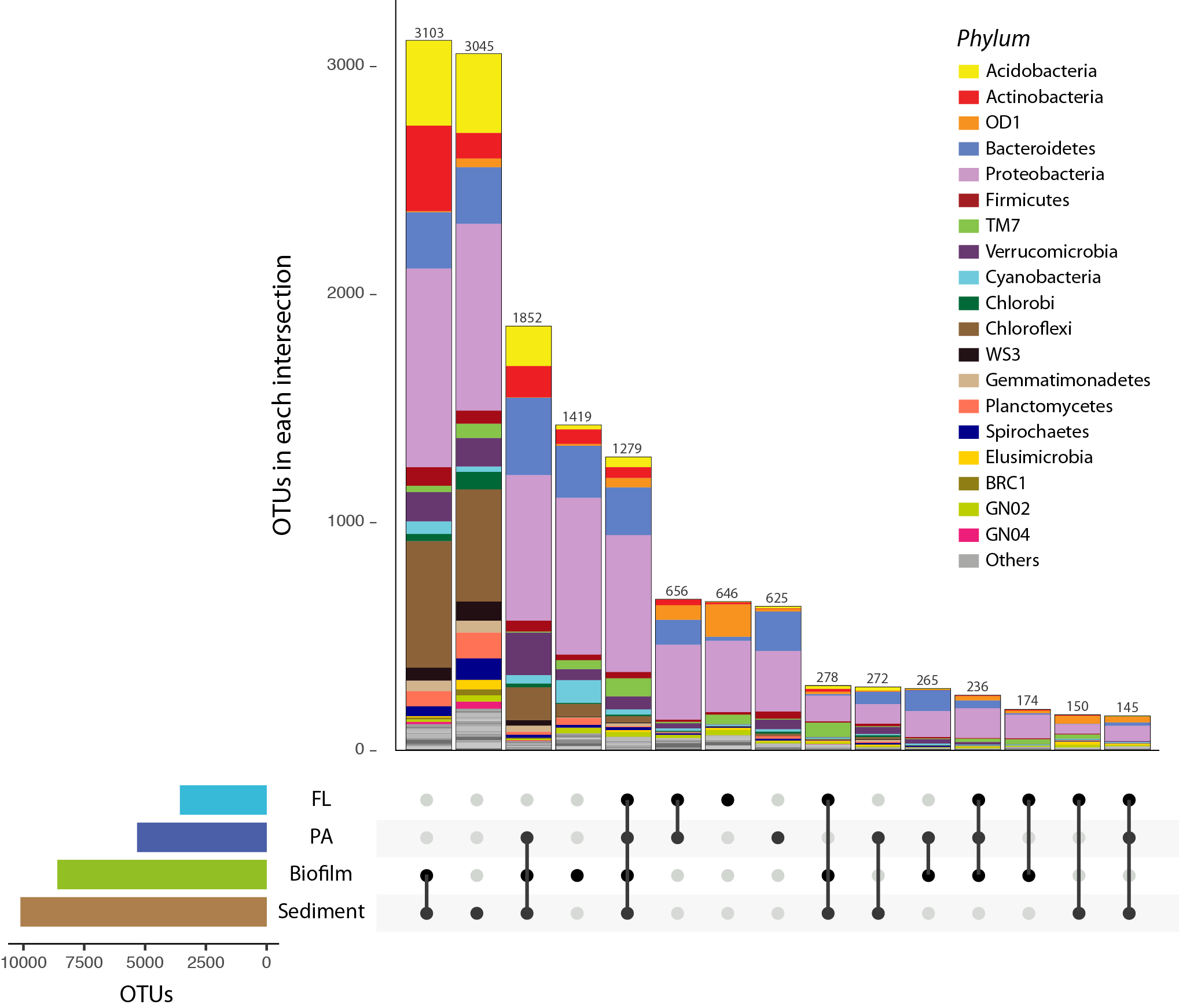


**Figure S1** Upset chart showing overlap in all OTUs identified in each habitat type. Numbers of OTUs shared between different sets of habitats are indicated in the top bar chart and the specific habitats in each set are indicated with solid points below the bar chart. Total number of OTUs for each habitat are indicated on the left. Figure generated using Upset R package (Lex et al., 2014). Do note that the bar chart is not showing relative abundances.
