# Supplementary material for: Contrasting community assembly processes structure lotic bacteria metacommunities along the river continuum
Source: Environ Microbiol. 2020 Dec 10;23(1):484–98. doi: 10.1111/1462-2920.15337 (PMC7898806; doi:10.1111/1462-2920.15337)
Supplement: Supplementary file 2 — Supplementary Table 1 PERMANOVA analyses produces a p‐value for significance and the R2 value, which indicates the amount of variation attributed to a specific treatment within a model. The R2 values indicate greater differences between the planktonic communities (Free‐Living and Particle‐Associated) and benthic communities (Biofilm and Sediment). [file EMI-23-484-s002.docx]

| *Pairwise Test* | *F* | *R2* | *p-value (Bonferroni corrected)* |
| --- | --- | --- | --- |
| FL x PA | 4.65089 | 0.174512 | 0.006 |
| FL x BF | 32.2717 | 0.594632 | 0.006 |
| FL x SE | 36.5868 | 0.624489 | 0.006 |
| PA x BF | 20.1341 | 0.477857 | 0.006 |
| PA x SE | 22.697 | 0.507796 | 0.006 |
| BF x SE | 6.61406 | 0.231147 | 0.006 |

**Supplementary Table 1** | PERMANOVA analyses produces a p-value for significance and the R2 value, which indicates the amount of variation attributed to a specific treatment within a model. The R2 values indicate greater differences between the planktonic communities (Free-Living and Particle-Associated) and benthic communities (Biofilm and Sediment).
