# Supplementary material for: Contrasting community assembly processes structure lotic bacteria metacommunities along the river continuum
Source: Environ Microbiol. 2020 Dec 10;23(1):484–98. doi: 10.1111/1462-2920.15337 (PMC7898806; doi:10.1111/1462-2920.15337)
Supplement: Supplementary file 3 — Supplementary Table 2 Tukey's HSD on diversity indices (Tukey multiple comparisons of means, 95% family‐wise confidence level) [file EMI-23-484-s003.docx]

*a. Shannon index of diversity*

| *Pairwise Comparison* | *Mean Difference* | *95% conf. intv.*  *Lower* | *95% conf. intv.*  *Upper* | *p-value adjusted* |
| --- | --- | --- | --- | --- |
| FL x PA | 0.80996585 | 0.32340490 | 1.2965268 | 0.00033309 |
| FL x BF | 2.49681250 | 2.01025154 | 2.9833734 | 0.00000000 |
| FL x SE | 3.12960126 | 2.64304031 | 3.6161622 | 0.00000000 |
| PA x BF | 1.68684665 | 1.20028570 | 2.1734076 | 0.00000000 |
| PA x SE | 2.31963542 | 1.83307446 | 2.8061964 | 0.00000000 |
| BF x SE | 0.63278877 | 0.14622782 | 1.1193497 | 0.00620084 |

*b. Pielou’s Evenness*

| *Pairwise Comparison* | *Mean Difference* | *95% conf. intv.*  *Lower* | *95% conf. intv.*  *Upper* | *p-value adjusted* |
| --- | --- | --- | --- | --- |
| FL x PA | 0.069624725 | 0.019617398 | 0.11963205 | 0.00306763 |
| FL x BF | 0.166028951 | 0.116021624 | 0.21603628 | 0.00000000 |
| FL x SE | 0.236913055 | 0.186905728 | 0.28692038 | 0.00000000 |
| PA x BF | 0.096404226 | 0.046396899 | 0.14641155 | 0.00003427 |
| PA x SE | 0.167288330 | 0.117281003 | 0.21729566 | 0.00000000 |
| BF x SE | 0.070884104 | 0.020876777 | 0.12089143 | 0.00251765 |

*c. Community heterogeneity*

| *Pairwise Comparison* | *Mean Difference* | *95% conf. intv.*  *Lower* | *95% conf. intv.*  *Upper* | *p-value adjusted* |
| --- | --- | --- | --- | --- |
| FL x PA | -0.030019469 | -0.058838896 | -0.0012000433 | 0.03832511 |
| FL x BF | -0.155089851 | -0.183909277 | -0.1262704249 | 0.00000000 |
| FL x SE | -0.176078434 | -0.204897860 | -0.1472590075 | 0.00000000 |
| PA x BF | -0.125070382 | -0.153889808 | -0.0962509554 | 0.00000000 |
| PA x SE | -0.146058964 | -0.174878390 | -0.1172395380 | 0.00000000 |
| BF x SE | -0.020988583 | -0.049808009 | 0.0078308435 | 0.22477011 |

**Supplementary Table 2** | Tukey’s HSD on diversity indice (Tukey multiple comparisons of means, 95% family-wise confidence level)
