# Supplementary material for: Contrasting community assembly processes structure lotic bacteria metacommunities along the river continuum
Source: Environ Microbiol. 2020 Dec 10;23(1):484–98. doi: 10.1111/1462-2920.15337 (PMC7898806; doi:10.1111/1462-2920.15337)
Supplement: Supplementary file 4 — Supplementary Table 3 Tukey's HSD on the differences in relative abundances of selected phyla between four different habitats (Tukey multiple comparisons of means, 95% family‐wise confidence level) [file EMI-23-484-s004.docx]

*a. Differences in relative abundances of Bacteroidetes between four habitats*

| *Pairwise Comparison* | *Mean Difference* | *95% conf. intv.*  *Lower* | *95% conf. intv.*  *Upper* | *p-value adjusted* |
| --- | --- | --- | --- | --- |
| FL x PA | 0.08551241 | 0.01157956 | 0.15944526 | 0.0176457 |
| FL x BF | **-0.2476179** | **-0.3215507** | **-0.173685** | **0.0000000** |
| FL x SE | **-0.2559899** | **-0.3299228** | **-0.1820571** | **0.0000000** |
| PA x BF | **-0.3331303** | **-0.4070631** | **-0.2591974** | **0.0000000** |
| PA x SE | **-0.3415023** | **-0.4154352** | **-0.2675695** | **0.0000000** |
| BF x SE | -0.0083721 | -0.0823049 | 0.06556078 | 0.9902659 |

*b. Differences in relative abundances of Cyanobacteria between four habitats*

| *Pairwise Comparison* | *Mean Difference* | *95% conf. intv.*  *Lower* | *95% conf. intv.*  *Upper* | *p-value adjusted* |
| --- | --- | --- | --- | --- |
| FL x PA | 0.005770691 | -0.03898891 | 0.05053029 | 0.9857874 |
| FL x BF | **0.130931223** | **0.08617162** | **0.17569082** | **0.0000000** |
| FL x SE | 0.014640988 | -0.03011861 | 0.05940059 | 0.8185428 |
| PA x BF | **0.125160532** | **0.08040093** | **0.16992013** | **0.0000000** |
| PA x SE | 0.008870296 | -0.03588930 | 0.05362990 | 0.9515487 |
| BF x SE | **-0.116290236** | **-0.16104984** | **-0.07153064** | **0.0000001** |

*c. Differences in relative abundances of OD1 between four habitats*

| *Pairwise Comparison* | *Mean Difference* | *95% conf. intv.*  *Lower* | *95% conf. intv.*  *Upper* | *p-value adjusted* |
| --- | --- | --- | --- | --- |
| FL x PA | **-0.0116203** | **-0.0165654** | **-0.0066752** | **0.0000008** |
| FL x BF | **-0.0133642** | **-0.0183093** | **-0.0084191** | **0.0000000** |
| FL x SE | **-0.0121039** | **-0.017049** | **-0.0071588** | **0.0000003** |
| PA x BF | -0.0017439 | -0.006689 | 0.00320117 | 0.7826967 |
| PA x SE | -0.0004836 | -0.0054287 | 0.00446146 | 0.9936711 |
| BF x SE | 0.0012603 | -0.0036848 | 0.00620539 | 0.9039626 |

**Supplementary Table 3** | Tukey’s HSD on the differences in relative abundances of selected phyla between four different habitats (Tukey multiple comparisons of means, 95% family-wise confidence level)
